# Vitamin D Levels and Uropathogen Distribution in Urinary Tract Infections: A Six-Year Retrospective Study from Cyprus

**DOI:** 10.3390/medicina62061113

**Published:** 2026-06-08

**Authors:** Hülya Arık, Mehtap Tınazlı, Kaya Süer

**Affiliations:** 1 Department of Infectious Diseases and Clinical Microbiology, School of Medicine, Near East University, 99138 Nicosia, Cyprus; kaya.suer@neu.edu.tr; 2 Department of Internal Medicine, School of Medicine, Near East University, 99138 Nicosia, Cyprus; mehtap.canbaz@hotmail.com

**Keywords:** vitamin D, urinary tract infection, uropathogen, seasonal variation, antimicrobial resistance, retrospective study, Cyprus

## Abstract

*Background and Objectives*: Urinary tract infections (UTIs) represent one of the most frequently encountered bacterial infections in clinical practice. Although vitamin D (vit D) is recognised for its immunomodulatory properties, its relationship with the spectrum of uropathogens remains unclear. This study investigated the distribution of UTI-causing pathogens in relation to serum vit D status and demographic variables including age, sex, season, and year of presentation at a tertiary hospital in the Eastern Mediterranean. *Materials and Methods*: A retrospective cross-sectional analysis was conducted on 942 adult patients with culture-confirmed UTIs at a university hospital in Cyprus between January 2019 and December 2024. Serum 25-hydroxyvitamin D [25(OH)D] levels were classified as deficient (≤20 ng/mL), insufficient (20–29.9 ng/mL), or sufficient (≥30 ng/mL) according to Turkish Endocrinology Society (TEMD) guidelines. Pathogen distribution was correlated with vit D category, sex, age group, season, and year using chi-square analysis and multivariable logistic regression. Timing of urine culture collection (at admission vs. more than 48 h after admission), catheter use, upper vs. lower urinary tract classification, and comorbidity data were recorded for each patient. *Results*: Escherichia coli was the most frequently isolated uropathogen (48.83%), followed by Klebsiella pneumoniae (18.79%) and Enterococcus faecalis (11.89%). No statistically significant association was found between vit D level and uropathogen type (*p* = 0.504). Infections were more prevalent in females (70.49%) and in patients aged over 70 years (56.26%). Vit D deficiency was present in 47.98% of the cohort. Catheter-derived specimens accounted for 35.1% of cultures. Upper tract infection was diagnosed in 233 patients (24.7%) and lower tract infection in 709 patients (75.3%). The most frequent comorbidities were hypertension (48.4%), diabetes mellitus (33.1%), and chronic kidney disease (21.0%); on multivariable analysis, diabetes mellitus (adjusted OR = 1.4) and chronic kidney disease (adjusted OR = 1.6) were independently associated with K. pneumoniae infection. In vit D-deficient patients, K. pneumoniae infection risk was significantly higher during winter in unadjusted analysis (OR = 1.8; 95% CI: 1.3–2.5) and remained elevated after multivariable adjustment (aOR = 1.6; 95% CI: 1.1–2.3). Vit D levels showed significant seasonal variation (*p* < 0.001), with lower values in winter (18.6 ng/mL) and higher values in summer (28.4 ng/mL). *Conclusions*: On multivariable analysis, no statistically significant association was found between vit D level and uropathogen species overall (χ^2^ = 13.291; *p* = 0.504); a seasonal interaction was observed between vit D deficiency and Klebsiella infections in winter. UTI risk was highest in elderly and female patients. These findings point to the need for considering seasonal and dietary factors in UTI management and call for prospective investigation.

## 1. Introduction

Urinary tract infections (UTIs) are among the most common bacterial infections encountered in both community and hospital settings, affecting an estimated 150 million individuals worldwide each year [1]. UTIs impose a considerable burden on healthcare systems in terms of antimicrobial consumption, diagnostic workload, and morbidity, particularly among women, elderly patients, and those with indwelling urinary catheters [2]. The spectrum of causative organisms varies according to whether the infection is community- or hospital-acquired, patient sex, age, immunological status, and geographic region [3]. Escherichia coli (E. coli) remains the predominant uropathogen, responsible for approximately 70–80% of uncomplicated UTIs in most settings [4]. Other clinically relevant organisms include Klebsiella pneumoniae, Proteus mirabilis, Enterococcus faecalis, and Pseudomonas aeruginosa, which are particularly prevalent in complicated or healthcare-associated infections [5].

Vitamin D (vit D) has attracted increasing attention in infectious disease research beyond its classical role in calcium and phosphorus homeostasis. The biologically active form of vit D, 1,25-dihydroxyvitamin D_3_ [1,25(OH)_2_D_3_], modulates both innate and adaptive immune responses through the vitamin D receptor (VDR) expressed on immune cells including macrophages, dendritic cells, and T lymphocytes [6,7]. Activation of the VDR signalling pathway enhances macrophage bactericidal function and induces the production of endogenous antimicrobial peptides, notably cathelicidin (LL-37) and β-defensins, which exert direct bactericidal activity against a range of Gram-negative and Gram-positive uropathogens [8]. Vit D also modulates the expression of toll-like receptors and regulates cytokine profiles, contributing to a balanced inflammatory response during infection [9].

Epidemiological studies have suggested an association between vit D deficiency and increased susceptibility to various infections, including respiratory tract infections, tuberculosis, and recurrent UTIs [10,11]. A meta-analysis by Martineau et al. demonstrated a protective effect of vit D supplementation against acute respiratory infections, particularly in individuals with baseline deficiency [12]. Regarding UTIs specifically, Nseir et al. reported significantly lower serum 25(OH)D levels in premenopausal women with recurrent UTIs compared with healthy controls [11]. However, the evidence remains inconsistent; a large randomised controlled trial involving 511 participants found no significant reduction in UTI incidence following five years of vit D supplementation [13]. These conflicting findings may be partially explained by heterogeneity in study populations, differences in vit D metabolism across age groups, variation in pathogen virulence, and the influence of unmeasured confounders such as seasonal sunlight exposure and dietary supplementation habits [14].

During the COVID-19 pandemic, public awareness and clinical interest in vit D supplementation increased markedly, driven by emerging evidence suggesting a potential protective role against SARS-CoV-2 infection severity [7,15]. This may have contributed to temporal changes in population-level vit D status in recent years, which should be considered when interpreting longitudinal studies of vit D and infection [15,16].

Anatomical factors confer a substantially higher baseline UTI risk in women, particularly in the context of sexual activity and postmenopausal oestrogen deficiency, which alters vaginal flora composition and promotes uropathogen colonisation [1,17,18]. In older adults, additional mechanisms including immunosenescence, neurogenic bladder dysfunction, increased catheter use, reduced prostatic secretions in men, and altered urogenital microbiota further increase vulnerability to both primary and recurrent UTIs [19,20,21]. The interplay between age-related immune decline and seasonal fluctuations in vit D levels adds further complexity to the relationship between host nutritional status and UTI epidemiology.

Cyprus, situated in the Eastern Mediterranean, provides a unique setting for investigating vit D–infection relationships due to its marked seasonal variation in solar ultraviolet B (UVB) radiation, with prolonged high-exposure summers and relatively mild but cloudy winters. This pronounced seasonality produces significant intra-annual fluctuations in population vit D levels, creating a natural model for examining how seasonal changes in vit D status may interact with pathogen distribution [22].

To date, no study has systematically examined the relationship between vit D status and the species-level distribution of uropathogens across a full annual cycle in the Eastern Mediterranean, nor has any investigation incorporated Infection Control Committee-based active surveillance data for this purpose. The present study addresses this gap. This study aimed to characterise the distribution of UTI-causing pathogens at a tertiary university hospital in Cyprus over a six-year period (2019–2024) and to evaluate the relationship between serum vit D levels and uropathogen type. Secondary objectives included examining the influence of patient sex, age group, year of presentation, and season on pathogen distribution, vit D levels, and the potential interaction between seasonal vit D fluctuations and specific pathogen frequencies.

## 2. Materials and Methods

### 2.1. Study Design and Setting

A retrospective cross-sectional study was conducted at University Hospital, a 350-bed tertiary referral centre in Nicosia, Cyprus, covering the period from 1 January 2019 to 31 December 2024. The hospital serves both local and referred patients from across the island and provides inpatient and outpatient infectious disease, internal medicine, urology, and geriatric care.

### 2.2. Patient Selection and Data Collection

Adult patients (≥18 years) who presented with a positive urine culture consistent with UTI (defined as ≥10^5^ colony-forming units per millilitre of a single uropathogen) and who had a serum 25(OH)D measurement recorded within the same clinical episode were eligible for inclusion. No standardised institutional protocol governed vitamin D testing during the study period; serum 25(OH)D was measured as part of routine clinical care at the discretion of the treating physician—for indications such as evaluation of bone health, fatigue, or general metabolic screening—rather than for the purposes of this study. A total of 4701 patient records were screened through the hospital information system (HIS). After applying exclusion criteria—age under 18 years, duplicate or repeat culture entries from the same infection episode, polymicrobial cultures, and absence of concurrent vit D data—942 patients were included in the final analysis.

Uropathogen data, clinical UTI classifications, and catheter-related information were obtained from the prospectively maintained records of the hospital’s Infection Control Committee (ICC), which conducts active surveillance of all healthcare-associated and community-acquired infections in accordance with national standards. Within this programme, urine culture results, upper versus lower urinary tract infection classification (pyelonephritis vs. cystitis), and the presence, type, and insertion date of urinary catheters are systematically documented for every patient using standardised surveillance forms. The use of ICC records as the primary data source ensured that UTI classification and catheter status were recorded prospectively and independently of the research team, minimising the risk of ascertainment bias. Additional clinical variables recorded at the time of UTI diagnosis included: (i) timing of urine culture collection relative to hospital admission (at admission vs. more than 48 h after admission) to distinguish community-acquired from healthcare-associated infections; (ii) presence of UTI-compatible symptoms (dysuria, urinary frequency, urgency, suprapubic pain, fever, or flank pain) documented in the patient’s medical records; (iii) classification of UTI as upper tract (pyelonephritis) or lower tract (cystitis) infection based on clinical presentation, imaging studies, and laboratory findings (elevated inflammatory markers, costovertebral angle tenderness), as recorded in ICC surveillance forms; (iv) timing of serum 25(OH)D measurement relative to the index urine culture, for which a window of ±7 days was applied as the eligibility criterion; and (v) the indication for serum vit D testing, which could not be systematically retrieved from retrospective medical records for all patients. As a surrogate, patients without a concurrent ICD-10 code for vitamin D deficiency, osteoporosis, or a related metabolic disorder at the time of testing were classified as undergoing routine metabolic screening (*n* = 412; 43.7%), while those with such a code were classified as clinically indicated (*n* = 530; 56.3%). The accuracy limits of this indirect classification are discussed in the Limitations section in the context of selection bias.

Demographic data (age, sex), clinical setting (inpatient vs. outpatient), microbiological culture results, serum vit D levels, and the date of clinical presentation were retrospectively extracted from the HIS. The method of urine specimen collection was documented for each patient: clean-catch midstream urine (CCMSU) specimens were obtained from cooperative outpatients and inpatients, while catheter-obtained urine specimens were collected from patients with indwelling urinary catheters. The proportion of catheter-derived versus clean-catch samples was analysed as a potential confounder in both pathogen distribution and vit D association analyses. Seasons were defined as: spring (March–May), summer (June–August), autumn (September–November), and winter (December–February).

### 2.3. Microbiological Methods

Mid-stream clean-catch urine specimens or catheter-obtained samples were processed using standard microbiological techniques. Specimens were inoculated onto Blood Agar (Becton, Dickinson and Company, Franklin Lakes, NJ, USA) (5% sheep blood) and Eosin Methylene Blue (EMB) Agar (Merck KGaA, Dramstadt, Germany) and incubated aerobically at 35 °C for 24–48 h. Colonies showing significant growth (≥10^5^ CFU/mL) were subcultured for identification. Organism identification to the species level and antimicrobial susceptibility testing were performed using the automated VITEK^®^ 2 Compact system (bioMérieux, Marcy-l’Étoile, France) with appropriate identification cards (GN for Gram-negatives, GP for Gram-positives). Susceptibility results were interpreted according to Clinical and Laboratory Standards Institute (CLSI) M100 (2023 edition) breakpoints [23]. Isolates were categorised into eight groups: E. coli, K. pneumoniae, E. faecalis, P. mirabilis, P. aeruginosa, Acinetobacter baumannii, Staphylococcus spp., and “Other” (comprising less frequently isolated species).

### 2.4. Vitamin D Assessment

Serum 25-hydroxyvitamin D [25(OH)D], the accepted biomarker of systemic vit D status, was measured using the Abbott Alinity i immunoassay analyser (Abott Laboratories, Abott Park, IL, USA) employing a chemiluminescent microparticle immunoassay (CMIA) technique. The assay has a reported measurement range of 3.4–156.0 ng/mL with an inter-assay coefficient of variation <10%. Patients were categorised into three groups according to the Turkish Society of Endocrinology and Metabolism (TEMD) guidelines: vit D deficient (≤20 ng/mL), insufficient (20.0–29.9 ng/mL), or sufficient (≥30 ng/mL). For subgroup analyses, severely deficient (<10 ng/mL) was also examined separately. Only 25(OH)D measurements obtained within ±7 days of the index urine culture were included in the primary analysis. This window was selected on the following basis: (i) the half-life of circulating 25(OH)D is approximately 2–3 weeks, ensuring that short-interval variation does not meaningfully alter vitamin D status classification; (ii) although 25(OH)D behaves as a negative acute-phase reactant through suppression of vitamin D binding protein, the magnitude of suppression in uncomplicated UTI is modest and unlikely to shift patients across established deficiency or sufficiency thresholds [24,25]; and (iii) this window provided an optimal balance between temporal validity and statistical power. The median interval between vit D measurement and urine culture collection was 2 days (IQR 1–4 days). The indirect ICD-10-based classification of vit D testing indication is described in Section 2.2. Sensitivity analyses comparing patients classified as routine screening versus clinically indicated testing were conducted to assess the consistency of primary findings in the presence of this potential selection bias.

### 2.5. Ethical Considerations

The study protocol was approved by the Near East University Institutional Ethics Committee (Project No: YDU/2024/126-1878). Given the retrospective design and the use of fully anonymised data extracted from the HIS, informed patient consent was waived in accordance with local regulatory standards. The study was conducted in compliance with the principles of the Declaration of Helsinki.

### 2.6. Statistical Analysis

All data were analysed using IBM SPSS Statistics version 27.0 (IBM Corporation, Armonk, NY, USA). Categorical variables are presented as frequencies (n) and percentages (%); continuous variables as mean ± standard deviation (SD). The normality of continuous data was assessed using the Kolmogorov–Smirnov test. Group comparisons for categorical variables were performed using the Chi-square (χ^2^) test or Fisher’s exact test where expected cell counts were <5. Continuous variables were compared between two groups using the independent samples *t*-test or Mann–Whitney U test, and among three or more groups using one-way ANOVA with Tukey’s post hoc test or the Kruskal–Wallis test as appropriate. Pearson’s correlation coefficient (r) was used to assess linear relationships between continuous variables.

Multivariable logistic regression was performed to estimate adjusted odds ratios (aORs) with 95% confidence intervals (CIs) for the association between vit D category and specific uropathogen infection risk, adjusting for potential confounders including age, sex, inpatient versus outpatient status, catheter-derived versus clean-catch urine samples, season, year of presentation, and presence of comorbidities (diabetes mellitus, chronic kidney disease, immunosuppression, and malignancy). Model fit was assessed using the Hosmer–Lemeshow goodness-of-fit test. Sensitivity analyses were conducted excluding patients with vit D measurements obtained for non-routine indications to assess selection bias. All tests were two-tailed, and a *p*-value < 0.05 was considered statistically significant.

## 3. Results

### 3.1. Patient Characteristics

The cohort comprised 942 patients: 664 female (70.49%) and 278 male (29.51%). The age distribution was as follows: 63 patients (6.69%) in the 18–30-year group, 91 (9.66%) in the 31–50-year group, 258 (27.39%) in the 51–70-year group, and 530 (56.26%) aged over 70 years. A total of 566 patients (60.08%) were hospitalised at the time of UTI diagnosis, while 376 (39.92%) were outpatients. The overall mean serum vit D was 23.31 ± 14.45 ng/mL. Vit D deficiency (≤20 ng/mL) was present in 452 patients (47.98%), insufficiency (20–29.9 ng/mL) in 253 (26.86%), and sufficiency (≥30 ng/mL) in 237 (25.16%). No significant sex difference in vit D level was observed (males: 23.01 ng/mL; females: 22.15 ng/mL; *p* = 0.672). The lowest vit D levels were recorded in the >70-year age group (21.5 ± 12.8 ng/mL), while the highest levels were found in the 18–30-year age group (28.3 ± 16.1 ng/mL); however, the correlation between age and vit D was not statistically significant (r = 0.055, *p* = 0.092).

Urine specimens were obtained by clean-catch midstream technique in 611 patients (64.9%) and by urinary catheter in 331 patients (35.1%). Among catheterised patients, 181 (19.2% of total cohort) had indwelling catheters placed >48 h prior to culture, consistent with healthcare-associated infection criteria.

UTI-compatible symptoms were documented in 797 patients (84.6%). Upper tract infection (pyelonephritis) was diagnosed in 233 patients (24.7%) and lower tract infection (cystitis) in 709 patients (75.3%). Symptom presence was not used as an inclusion criterion; asymptomatic bacteriuria and atypically presenting cases—particularly elderly and hospitalised patients presenting with non-specific features such as confusion or malaise rather than classic UTI symptoms—were intentionally retained in the analysis, as the primary aim was to evaluate the relationship between vit D status and uropathogen distribution independently of symptom status.

Comorbidity distribution across vit D categories was also examined among the 847 patients with available comorbidity records, with within-category percentages expressed relative to each vit D subgroup.

#### Comorbidity and Pathogen Association

In multivariable logistic regression adjusting for age, sex, inpatient status, catheter-derived samples, season, and year, vit D deficiency (≤20 ng/mL) remained associated with K. pneumoniae infection during winter (adjusted OR = 1.6; 95% CI: 1.1–2.3; *p* = 0.012). However, this association was attenuated compared with the unadjusted analysis (OR = 1.8; 95% CI: 1.3–2.5), suggesting partial confounding by comorbidity burden and healthcare exposure. Diabetes mellitus (adjusted OR = 1.4; 95% CI: 1.1–1.9; *p* = 0.018) and chronic kidney disease (adjusted OR = 1.6; 95% CI: 1.2–2.2; *p* = 0.003) were independently associated with K. pneumoniae infection. The Hosmer–Lemeshow test indicated adequate model fit (*p* = 0.412). In sensitivity analyses restricted to patients with vit D measurements obtained for routine metabolic screening (*n* = 412), the association between vit D deficiency and winter K. pneumoniae infection remained consistent (aOR = 1.5; 95% CI: 1.0–2.2; *p* = 0.041), indicating that selection bias related to vit D testing indication did not materially alter the primary finding.

### 3.2. Overall Pathogen Distribution and Sex Differences

**Figure 1:**
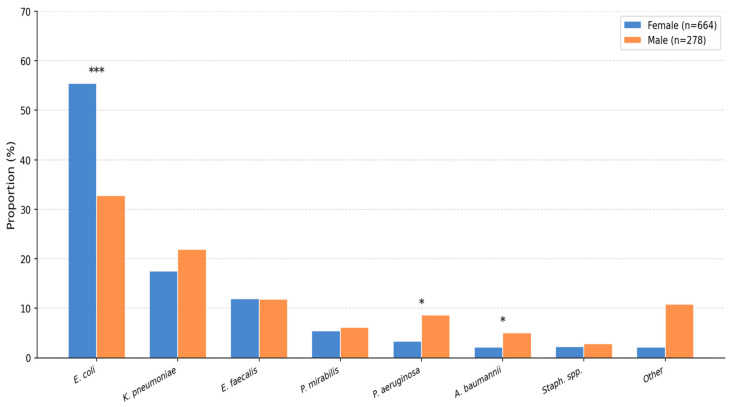
Sex-based distribution of uropathogens among 942 patients with culture-confirmed UTIs. * *p* < 0.05; *** *p* < 0.001 (Chi-square test).

Escherichia coli was the most frequently isolated pathogen (460/942; 48.83%), followed by K. pneumoniae (177/942; 18.79%), E. faecalis (112/942; 11.89%), P. mirabilis (53/942; 5.63%), P. aeruginosa (46/942; 4.88%), A. baumannii (28/942; 2.97%), Staphylococcus spp. (23/942; 2.44%), and other organisms (43/942; 4.56%). Significant sex-based differences were observed: E. coli was more prevalent in females than males (55.42% in females vs. 32.73% in males; *p* < 0.001). K. pneumoniae showed a numerically higher proportion in males than females (21.94% in males vs. 17.47% in females), though this difference did not reach statistical significance (*p* = 0.089). Conversely, P. aeruginosa (3.31% in females vs. 8.63% in males) and A. baumannii (2.11% in females vs. 5.04% in males) were significantly more frequent in males (*p* < 0.05). Sex-based pathogen distributions are illustrated in Figure 1.

### 3.3. Vitamin D Level and Pathogen Type

Chi-square analysis revealed no statistically significant association between vit D category and uropathogen species (χ^2^ = 13.291, *p* = 0.504). E. coli was the predominant isolate across all vit D categories: <10 ng/mL (48.1%), 10–29.99 ng/mL (48.3%), and ≥30 ng/mL (50.6%). Although A. baumannii (2.5%) and Staphylococcus spp. (3.1%) were numerically less common in the severely deficient group (<10 ng/mL), these differences did not reach statistical significance (*p* > 0.05). The complete pathogen distribution by vit D category is presented in Table 1.

### 3.4. Age-Related Differences in Pathogen Distribution

The proportion of E. coli infections decreased significantly with advancing age (60.3% in the 18–30-year group vs. 44.9% in those >70 years; *p* = 0.021), while E. faecalis (4.8% to 15.5%; *p* = 0.002) and P. aeruginosa (1.6% to 6.6%; *p* = 0.038) showed a significant increase with age. Patients aged >70 years demonstrated the highest rates of E. faecalis (15.5%) and P. aeruginosa (6.6%) compared with all other age groups (*p* < 0.05). No significant age-related variation was observed for K. pneumoniae, P. mirabilis, A. baumannii, or Staphylococcus spp. (all *p* > 0.05). Age-stratified data are presented in Table 2.

### 3.5. Annual and Seasonal Trends

**Figure 2:**
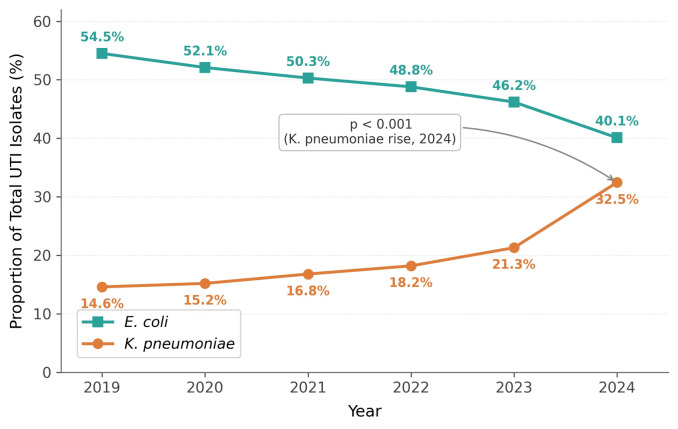
Annual trends in E. coli and K. pneumoniae proportions among UTI isolates (2019–2024). The sharp increase in K. pneumoniae in 2024 was statistically significant (*p* < 0.001).

UTI episodes were most frequently documented in 2019 (178/942; 18.90%) and 2022 (170/942; 18.05%). A marked rise in K. pneumoniae was observed in 2024, reaching 32.45% of that year’s isolates (*p* < 0.001), while E. coli showed a corresponding decline from 54.5% in 2019 to 40.1% in 2024. These diverging annual trends are shown in Figure 2. K. pneumoniae was isolated in 24.0% of hospitalised patients (n = 136) and 10.9% of outpatients (n = 41). Mean vit D levels in 2023 and 2024 were significantly higher than in preceding years (*p* < 0.001), possibly reflecting increased supplementation practices.

Seasonal evaluation revealed that E. coli remained the predominant pathogen across all seasons (range: 40–55%), with the highest prevalence during summer (June–August; 52.3%). K. pneumoniae infections were significantly more frequent during winter (December–February; 24.7%) compared with summer (18.1%; *p* < 0.05). E. faecalis was more frequently isolated during autumn and winter (14.6%) compared with summer (9.8%). P. aeruginosa also demonstrated a seasonal pattern, with higher incidence in summer and autumn (6.1%) compared with winter (3.9%).

**Figure 3:**
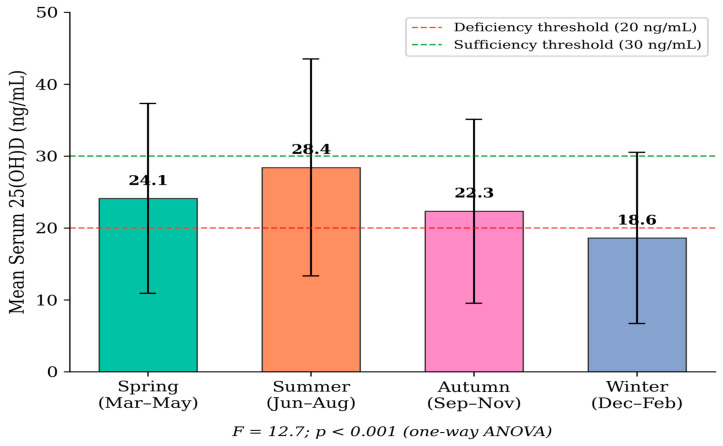
Seasonal variation in mean serum 25(OH)D levels (ng/mL). Dashed lines indicate deficiency (≤20 ng/mL) and sufficiency (≥30 ng/mL) thresholds.

Mean serum vit D was highest in summer (28.4 ng/mL) and lowest in winter (18.6 ng/mL). The seasonal variation was statistically significant (F = 12.7; *p* < 0.001; Figure 3). Vit D deficiency prevalence was 58.0% in winter versus 32.1% in summer (*p* < 0.001). A positive correlation was observed between vit D levels and sun-rich months (May–September) (r = 0.39; *p* = 0.002).

### 3.6. Vitamin D, Season, and Pathogen Interaction

**Figure 4:**
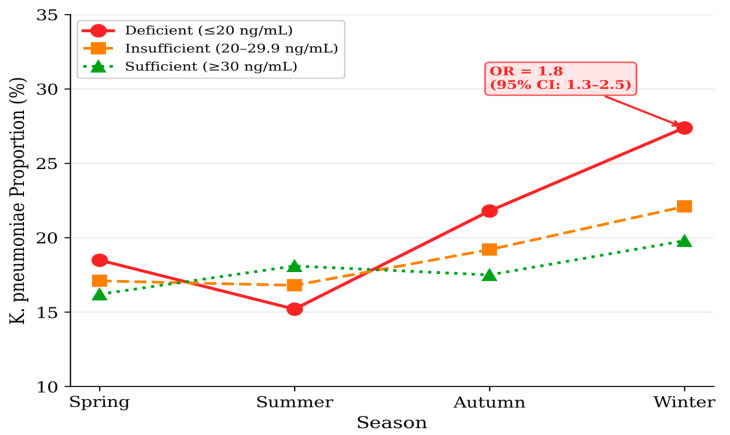
Seasonal K. pneumoniae proportions stratified by vitamin D status. Vit D-deficient patients (≤20 ng/mL) showed a sharp winter increase (OR = 1.8; 95% CI: 1.3–2.5; aOR = 1.6; 95% CI: 1.1–2.3 after multivariable adjustment).

Among vit D-deficient patients (<20 ng/mL), the risk of K. pneumoniae infection was 1.8 times higher in winter than in summer in unadjusted analysis (OR = 1.8; 95% CI: 1.3–2.5; Figure 4). After multivariable adjustment for age, sex, inpatient status, catheter-derived samples, comorbidities, and year, this association was attenuated but remained statistically significant (aOR = 1.6; 95% CI: 1.1–2.3; *p* = 0.012). E. coli infections were more frequent in the same group during summer (56.1%). Among patients with sufficient vit D levels (≥30 ng/mL), P. aeruginosa incidence was significantly lower in summer (*p* = 0.03). In patients aged >70 years, vit D deficiency (63.4%) and Enterococcus infections (16.2%) co-peaked during winter. In contrast, in young adults (18–30 years), vit D sufficiency was relatively high (70.5%) during summer and E. coli remained the most prevalent pathogen (61.3%). These findings suggest that infection patterns are influenced by the interaction between seasonal vit D fluctuations and age-related immune factors.

## 4. Discussion

The central finding of this study is the absence of a statistically significant association between serum vit D level and uropathogen type (χ^2^ = 13.291, *p* = 0.504) in unadjusted analysis. After multivariable adjustment for age, sex, comorbidities, healthcare exposure, and specimen collection method, this null finding was confirmed. Despite established evidence that vit D stimulates cathelicidin synthesis in macrophages and upregulates defensin expression in uroepithelial cells [6,8,9], our results indicate that vit D status alone does not determine which pathogen species causes a UTI. This finding aligns with the five-year randomised trial by Jorde et al. [13], which reported no reduction in UTI incidence with high-dose vit D supplementation, and with the observation by Kara et al. [10] that the vit D–UTI association is inconsistent when confounders are accounted for.

The observed seasonal interaction between vit D deficiency and K. pneumoniae infection during winter (unadjusted OR = 1.8) requires cautious interpretation. While this finding is biologically plausible given the immunomodulatory role of vit D in cathelicidin-mediated innate immunity, several unmeasured confounders may have contributed to this association.

First, data on vit D supplementation history, dietary intake, and sunlight exposure habits could not be systematically captured; these factors could independently influence both baseline vit D status and general health-seeking behaviour. Additionally, the proportion of catheter-derived urine samples (35.1%) differed between hospitalised and outpatient populations, and catheterisation itself is a known risk factor for K. pneumoniae and other healthcare-associated uropathogens. Although we adjusted for inpatient status in multivariable analyses, the catheterisation rate within the hospitalised subgroup was not uniformly distributed across seasons or vit D categories.

Comorbidity burden—including diabetes mellitus, chronic kidney disease, and immunosuppressive therapy—was higher among vit D-deficient patients (Table 3) and may have confounded the association between vit D status and pathogen type. Our multivariable analysis demonstrated attenuation of the K. pneumoniae–vit D association after adjusting for these factors (adjusted OR = 1.6 vs. unadjusted OR = 1.8), suggesting that comorbidities partially mediate this relationship. Although vit D measurement was restricted to a ±7-day window around the index culture and the median interval was only 2 days (IQR 1–4 days), the possibility that some measurements captured an acute-phase reduction in 25(OH)D cannot be entirely excluded. However, as 25(OH)D behaves as a negative acute-phase reactant primarily through suppression of vitamin D binding protein, the magnitude of this effect in uncomplicated UTI is substantially smaller than in severe systemic inflammation, and is unlikely to have shifted the majority of patients across established deficiency or sufficiency thresholds.

Finally, the indication for vit D testing (routine screening vs. clinical suspicion of deficiency) introduced potential selection bias. Patients tested for specific clinical indications may have had systematically different vit D levels and infection risk profiles compared with those undergoing routine screening. Future prospective studies should incorporate standardised vit D measurement protocols, detailed supplementation histories, and comprehensive comorbidity assessment to clarify the independent contribution of vit D status to uropathogen-specific infection risk. These findings also carry direct clinical implications. The convergence of vit D deficiency and K. pneumoniae infection risk during winter—particularly in elderly and hospitalised patients—suggests that targeted seasonal supplementation strategies merit evaluation in prospective interventional trials. Although our study cannot establish causality, the magnitude of the adjusted seasonal interaction (aOR = 1.6) and its biological plausibility support the design of such trials. Furthermore, the sharp rise in K. pneumoniae in 2024 has immediate stewardship implications: empirical regimens for complicated UTIs in this region should be reviewed against current local resistance profiles, and real-time resistance surveillance should be integrated into institutional antimicrobial stewardship programmes.

Several factors may account for the null result. The heterogeneous nature of the study population—encompassing patients with varying comorbidity profiles, unknown supplementation histories, and different durations between vit D measurement and urine culture—may have diluted any true association. In addition, the age-related decline in renal 1α-hydroxylase activity limits conversion of circulating 25(OH)D to the bioactive metabolite 1,25(OH)_2_D_3_, even when systemic 25(OH)D levels appear adequate [14]. This concept, termed “functional vit D deficiency,” could partially explain why elderly patients in our cohort (who had the lowest vit D levels at 21.5 ± 12.8 ng/mL) did not show a clear dose–response relationship between vit D status and pathogen type. The retrospective design also precluded measurement of local renal or urothelial vit D activation, which may be more relevant than circulating levels for antimicrobial peptide induction in the urinary tract. Future studies incorporating urinary cathelicidin levels or VDR polymorphism analysis may provide further mechanistic insight [14].

The predominance of E. coli (48.83%) across all demographic and vit D subgroups is consistent with established surveillance data from Mediterranean and European settings [1,3,4]. Although the frequency is somewhat lower than the 70–80% typically reported for uncomplicated community-acquired UTIs, this likely reflects the high proportion of hospitalised patients in our cohort (60.1%), among whom non-E. coli uropathogens are more prevalent. The age-related shift from E. coli dominance toward increased proportions of E. faecalis (*p* = 0.002) and P. aeruginosa (*p* = 0.038) in elderly patients is clinically significant and reflects the transition from simple community-acquired cystitis to complicated UTI associated with catheterisation, urological instrumentation, impaired host defences, and the biofilm-forming capacity of these organisms [19,20,26]. The higher proportions of P. aeruginosa (8.63%) and A. baumannii (5.04%) in males are consistent with healthcare-associated infections frequently linked to invasive urological procedures and prolonged hospitalisation [27,28].

The sharp rise in K. pneumoniae in 2024, reaching 32.45% of that year’s isolates (*p* < 0.001), is a finding of particular concern. This trend aligns with the growing burden of extended-spectrum β-lactamase (ESBL)-producing and carbapenem-resistant K. pneumoniae strains reported across Southern Europe and the Eastern Mediterranean [29,30,31]. Bağkur et al. previously modelled that ESBL-producing K. pneumoniae prevalence at this institution would continue to rise unless proactive antimicrobial stewardship measures were implemented [32]. Our six-year empirical surveillance data now provide direct observational confirmation of that projection, reinforcing the urgency of local resistance surveillance, antimicrobial susceptibility monitoring, and targeted stewardship interventions in the Eastern Mediterranean region. Although resistance genotyping was outside the scope of the present study, this represents a priority for future analyses integrating molecular epidemiology with clinical surveillance data.

The observed annual increase in population vit D levels during 2023–2024 compared with earlier years (*p* < 0.001) deserves attention. This temporal trend may reflect the widespread increase in vit D supplementation practices that followed heightened public and clinical awareness during the COVID-19 pandemic, when multiple studies suggested that adequate vit D status might confer protection against SARS-CoV-2 infection severity [7,15,16]. This secular trend represents a potential confounder in longitudinal studies of vit D and infection and calls for time-stratified analysis.

The seasonal interaction between vit D deficiency and K. pneumoniae risk in winter (unadjusted OR = 1.8; 95% CI: 1.3–2.5; adjusted OR = 1.6; 95% CI: 1.1–2.3) is an important secondary finding that requires prospective investigation. During winter, reduced solar UVB exposure leads to decreased cutaneous vit D synthesis and correspondingly lower serum levels (18.6 ng/mL in our cohort), while prolonged indoor confinement may increase transmission risk of nosocomial pathogens such as Klebsiella and Enterococcus [20,22]. Vit D deficiency may further impair innate immune defences by downregulating cathelicidin expression, creating a “double-hit” scenario in which both environmental and immunological factors converge to increase susceptibility to these organisms [6,8].

The seasonal peak of E. coli in summer (52.3%) may be attributable to environmental temperature and humidity effects that promote bacterial replication, as well as increased fluid loss and reduced urine concentration that may impair urinary tract defences [18]. The co-occurrence of vit D deficiency (63.4%) and Enterococcus infections (16.2%) during winter in the >70-year age group points to the convergence of age-related immunosenescence, seasonal nutritional deficiency, and the propensity for complicated UTIs in elderly patients.

### Strengths and Limitations

This study has several strengths: a large cohort (n = 942) that enhances statistical power and reliability; a six-year observation window (2019–2024) enabling evaluation of both seasonal and annual trends; standardised microbiological methods with automated identification; multivariable adjustment for comorbidities, catheter use, and vit D testing indication; and matched vit D measurements in a Mediterranean population with marked seasonal sunlight variation, which provides a natural gradient for examining vit D–infection interactions.

However, several limitations must be acknowledged. The retrospective, single-centre design limits causal inference and generalisability. Data on vit D supplementation history, dietary intake, and individual sunlight exposure patterns were not systematically available, and these unmeasured variables could independently influence vit D status and infection susceptibility. Comorbidity burden—including diabetes mellitus, chronic kidney disease, and immunosuppressive therapy—was more prevalent among vit D-deficient patients, which may have partially confounded observed associations between vit D and pathogen-specific infection risk. The proportion of catheter-derived urine samples (35.1%) varied across patient subgroups and seasons and is a known risk factor for healthcare-associated uropathogens; residual confounding by catheterisation within the hospitalised subgroup cannot be excluded. The indication for vit D testing was approximated using concurrent ICD-10 diagnostic codes rather than direct retrieval from clinical notes, classifying 43.7% of patients as routine screening and 56.3% as clinically indicated. This indirect classification may not accurately reflect the true indication in all cases, as some clinical indications may not have been assigned a separate diagnostic code on the day of testing; the resulting misclassification could introduce systematic differences in baseline vit D levels and health profiles between groups, representing a source of selection bias that should be interpreted accordingly. Regarding temporal confounding, although vit D measurement was restricted to a ±7-day window around the index culture (verified from the hospital information system (HIS)) and the median interval was 2 days (IQR 1–4 days), 25(OH)D is a negative acute-phase reactant whose serum concentration can decrease transiently during active infection through suppression of vitamin D binding protein; this effect, while modest in uncomplicated UTI, cannot be entirely excluded in patients with more pronounced inflammatory responses. Microbiological characterisation did not include resistance genotyping, virulence factor profiling, or biofilm assessment. Recurrent UTI episodes from the same patient were excluded, precluding assessment of within-patient longitudinal trends.

Future prospective cohort studies incorporating molecular microbiological techniques, VDR polymorphism analysis, urinary cathelicidin measurements, standardised vit D sampling protocols, detailed supplementation histories, and data on the gut and urogenital microbiome composition [33,34,35] are warranted to elucidate the mechanistic role of vit D in UTI pathogenesis and to determine whether seasonal vit D supplementation has a protective effect against specific uropathogen infections.

## 5. Conclusions

No statistically significant association was identified between serum vit D level and uropathogen species in this six-year cohort from a tertiary hospital in Cyprus after adjusting for age, sex, comorbidities, healthcare exposure, and specimen collection method. UTI risk was highest in elderly (≥70 years) and female patients. A seasonal interaction between vit D deficiency and K. pneumoniae infection during winter was observed (unadjusted OR = 1.8; 95% CI: 1.3–2.5); however, this association was attenuated after multivariable adjustment (adjusted OR = 1.6; 95% CI: 1.1–2.3) and may be partially confounded by comorbidity burden, catheterisation rates, and vit D testing selection bias, warranting cautious interpretation and prospective evaluation. The sharp rise in K. pneumoniae in 2024 points to the need for continued antimicrobial surveillance and stewardship in the Eastern Mediterranean region. These data support the consideration of seasonal, nutritional, and comorbidity factors in UTI risk stratification and clinical management. Prospective cohort studies with standardised vit D assessment, detailed supplementation histories, and comprehensive comorbidity data are needed to determine whether targeted seasonal supplementation can independently modify uropathogen-specific infection risk.

## Figures and Tables

**Table 1:** Uropathogen distribution by serum vitamin D category (*n* = 942). Chi-square, χ^2^ = 13.291, *p* = 0.504.

Vit D (ng/mL)	n	E. coli	K. pneum.	E. faecalis	P. mirabilis	P. aerug.	A. baum.	Staph.	Other
<10	160	77 (48.1%)	23 (14.4%)	28 (17.5%)	9 (5.6%)	6 (3.8%)	4 (2.5%)	5 (3.1%)	8 (5.0%)
10–29.99	545	263 (48.3%)	101 (18.5%)	59 (10.8%)	32 (5.9%)	31 (5.7%)	19 (3.5%)	13 (2.4%)	27 (5.0%)
≥30	237	120 (50.6%)	53 (22.4%)	25 (10.5%)	12 (5.1%)	9 (3.8%)	5 (2.1%)	5 (2.1%)	8 (3.4%)
Total	942	460 (48.8%)	177 (18.8%)	112 (11.9%)	53 (5.6%)	46 (4.9%)	28 (3.0%)	23 (2.4%)	43 (4.6%)

**Table 2:** Age-stratified uropathogen distribution (*n* = 942). Chi-square test; *p* < 0.05 considered statistically significant.

Pathogen	18–30 yr (*n* = 63)	31–50 yr (*n* = 91)	51–70 yr (*n* = 258)	>70 yr (*n* = 530)	Total (*n* = 942)	*p* Value
E. coli	38 (60.3%)	52 (57.1%)	132 (51.2%)	238 (44.9%)	460 (48.8%)	0.021
K. pneumoniae	8 (12.7%)	14 (15.4%)	48 (18.6%)	107 (20.2%)	177 (18.8%)	0.185
E. faecalis	3 (4.8%)	5 (5.5%)	22 (8.5%)	82 (15.5%)	112 (11.9%)	0.002
P. mirabilis	4 (6.3%)	6 (6.6%)	15 (5.8%)	28 (5.3%)	53 (5.6%)	0.892
P. aeruginosa	1 (1.6%)	2 (2.2%)	8 (3.1%)	35 (6.6%)	46 (4.9%)	0.038
A. baumannii	1 (1.6%)	3 (3.3%)	7 (2.7%)	17 (3.2%)	28 (3.0%)	0.812
Staph. spp.	2 (3.2%)	3 (3.3%)	6 (2.3%)	12 (2.3%)	23 (2.4%)	0.901
Other	6 (9.5%)	6 (6.6%)	20 (7.8%)	11 (2.1%)	43 (4.6%)	0.342

**Table 3:** Comorbidity distribution by serum vitamin D category (n = 847). Chi-square test; *p* < 0.05 considered statistically significant.

Comorbidity	Vit D <10 ng/mL	Vit D 10–29.99 ng/mL	Vit D ≥30 ng/mL	Total	*p*-Value
Diabetes mellitus	58 (36.3%)	178 (32.7%)	76 (32.1%)	312	0.742
Chronic kidney disease	42 (26.3%)	112 (20.6%)	44 (18.6%)	198	0.198
Hypertension	89 (55.6%)	234 (42.9%)	133 (56.1%)	456	0.003
Immunosuppression	15 (9.4%)	38 (7.0%)	14 (5.9%)	67	0.512
Malignancy	19 (11.9%)	42 (7.7%)	28 (11.8%)	89	0.189

## Data Availability

The data presented in this study are available on request from the corresponding author. The data are not publicly available due to patient confidentiality requirements.
